# Note on Cumulative Action of Medicines

**Published:** 1863-01

**Authors:** Alexander Fleming

**Affiliations:** Fellow of the Royal College of Physicians, London, &c.


### NOTE ON CUMULATIVE ACTION OF MEDICINES.

By ALEXANDER FLEMING, M. D., Fellow of the Royal College of Physicians, London, &c.

In my lectures on therapeutics, I have found it useful to the student to describe three modes of exhibiting medicines, in relation to the interval between the doses: the *simple*, where the second dose is not given until the action of the first has completely subsided; the *sustained*, where the doses are repeated at such intervals as to keep up without increase or diminution the required degree of physiological action, as in the use of wine; and, thirdly, the *cumulative.*

Although the term cumulative action is much used, it is often wrongly applied, and its meaning is, at all times, vague and uncertain. It is commonly employed to denote the exhibition of a medicine for some days, in small and repeated doses, without marked effect, when suddenly and unexpectedly violent, and, it may be, dangerous symptoms of its action are developed. The successive doses are supposed to remain in some obscure and unexplained way quiescent in the blood, until the so-called cumulative action is manifested. The administration of digitalis and strychnia are cited as affording examples. It is said that digitalis may be given continuously for several days, and apparently without effect, when suddenly a feeble, irregular pulse, fainting and cold sweats, usher in the poisonous action of the drug. In like manner, successive pills of strychnia may be taken and remain inert, when unexpectedly and suddenly severe tetanic symptoms supervene.

Now the sudden eruption of alarming symptoms during the continuous use of these medicines can be satisfactorily explained without reference to any mysterious agency. In the case of strychnia there is no cumulative action, but simply an example of the nonsolution and retention of the medicine in the stomach and bowels. It is observed only when the medicine is given in pill. This alkaloid is hard of solution in the gastric fluid, and one, two, three, or more pills are apt to remain undissolved and accumulate in the stomach or bowels. Suddenly, from some change in the patient, there is an abundant flow of gastric juice, and all the pills are simultaneously rendered soluble and active. This apparent cumulation of strychnia is never observed when it is given in solution. In the exhibition of digitalis, on the contrary, as I shall presently explain, we have an example of true cumulative action but imperfectly observed and understood.

I think it would be more correct to restrict the term *cumulation*, or *cumulative action*, to denote exclusively the gradual increase of physiological action from the successive exhibition of equal doses. When a second dose is given before the effects of the first have passed away, we add to what remains of the action of the first the full operation of the second, and so on with the third and subsequent doses until, finally, the sum of effects exceeds the limits of medicinal, and passes into those of poisonous, action.

In the exhibition of mercury, arsenic, aconite, digitalis, and other medicines, we adopt the cumulative mode, because it is safer and more efficient. The tolerance of these medicines varies so much that no physician can say what amount of any one of them is required to produce a given degree of physiological action. He might exceed the proper dose and cause danger. But by the cumulative addition of the effects of successive doses in the manner described, we advance cautiously and safely to the required degree of action, and the symptoms are more under control. The interval between the doses is determined in each medicine by the duration of its action. Between successive doses of mercury it may be twelve or even twenty-four hours, while, to secure the cumulative action of digitalis, it should not exceed four to eight hours. Our knowledge here is imperfect in respect to many drugs, and cannot be reduced to rules.

The cumulative mode of exhibition is most necessary with sedatives, as aconite and digitalis; and in using them the pulse must be carefully observed, for it is an important fact, in connection with sedation, that the circulation may be lowered to a remarkable degree without the patient being conscious of, or showing any material change in his other functions. But carry the depression just a little further, and the heart's action is suddenly and seriously embarrassed, and the patient has fainting, cold sweats, and the sense of impending dissolution. Now, the early effects on the pulse of the cumulative depression of digitalis are apt to be overlooked, and the belief obtains, as already stated, that the first doses produce no effect whatever; but this is an error. I have often watched the cumulative use both of digitalis and of aconite, and have never failed to detect depression of the circulation from the early doses, and its subsequent gradual increase. I should add that, as a sedative, I am careful to give digitalis so as to secure its prompt and easy absorption, and to avoid its local irritant effect on the stomach, which complicates the general symptoms of the medicine.

On the other hand, in the administration of atropia and strychnia it is no less important to avoid cumulation. Their use often extends over a considerable time, and it is safer to use the *simple* mode of exhibition, and to give the doses at such intervals that the action of the first has entirely subsided before the second is taken. Nor is there any danger with these drugs in inducing the required degree of medicinal action with one dose, provided its amount be determined by careful trials, commencing with small and advancing gradually to larger doses. For example, I give atropia thus,—10 minims (containing 1-60 of a grain) of a solution are exhibited once daily, and the dose is increased daily by 2 or 4 minims until I obtain the required degree of atropism. The action of one dose endures sixteen or eighteen hours, but ceases before the next is given and there is no cumulation, which it is safer to avoid, especially as the use of atropia is often continued for several weeks.

There is another order of physiological effects, sometimes named cumulative, which require notice here. They have been observed to follow the use of alcohol and aconite. I refer to the wakefulness, tremor, and exhaustion (delirium tremens), from prolonged excess, symptoms quite different from the ordinary stimulant and narcotic action of spirit, but distinctly traceable to its *continued use.* In the same manner, I have observed in patients who have been taking aconite in full doses for a lengthened period, that ultimately they have become affected with general tremors, severe pain in the head and eyeballs, constant lachrymation, intense photophobia, heat of skin, quick pulse and great restlessness, symptoms which, while very different from the ordinary sedative action of aconite, were clearly attributable to its long-continued employment. In the cases where I noticed these results, the aconite being discontinued, the symptoms, which were by no means alarming, subsided in a day or two. Effects allied in nature to this action have also been traced to tobacco and to mercury.

In my lectures, to prevent the student confounding these effects with cumulation, I found it needful to distinguish them by the name of *sequel* action. At present, the study of the sequel action of medicines does not offer much interest in the way of practical application; but it might at a future time acquire more importance. For example, one of the patients who exhibited the sequel action of aconite had been long a confirmed intermittent drunkard. From the time that he presented these symptoms the craving for spirit, previously so irresistible, never returned, and he continued to lead a sober life for at least four years while I knew him. Meantime it is well not to confound together things essentially distinct. The sequel action is due to the continued exhibition, and is a direct operation of the medicine, and must not itself be confounded with the symptoms which arise from the sudden suspension of a drug, such, for example, as opium, to which the body has become habituated. The extreme nervous prostration and excessive perspiration, urination and diarrhœa, which supervene on the sudden withdrawal of the habitual supply of this narcotic, are a good example of one of the forms of medicinal *reaction*. The cumulative and sequel actions are both phenomena of the forward, the reaction of the backward, swing of the physiological pendulum.

In relation to strychnia, I have already referred to the error of confounding true cumulative action with the accumulation of medicine in the bowels. This evil happens with solid medicines, as caustic magnesia, strychnia, and calomel, which require the intervention of the gastro-intestinal secretions for their solution and absorption. If the dose exceed the solvent power of the fluids, or these are deficient in quantity, then a portion of all the medicine remains undissolved, and is either expelled quickly by stool or lodges for a time in the bowels. If, while there, the visceral fluids become more abundant, it may be suddenly dissolved and by its absorption give rise to severe effects. I could cite many examples of this, but I have said enough to make evident the distinction between cumulation and the effects produced by the sudden solution and activity of a solid drug which has accumulated in the bowels.—*Boston Medical Journal.*

